# Supporting the mental health of children with speech, language and communication needs: The views and experiences of parents

**DOI:** 10.1177/23969415221101137

**Published:** 2022-05-29

**Authors:** Hannah Hobson, Mya Kalsi, Louise Cotton, Melanie Forster, Umar Toseeb

**Affiliations:** Department of Psychology, 8748 University of York, York, UK; Department of Psychology, 8748 University of York, York, UK; Faculty of Education and Health and Human Sciences, 411444 University of Greenwich, Kent, UK; Department of Psychology, 8748 University of York, York, UK; Department of Education, 8748 University of York, York, UK

**Keywords:** developmental language impairment (DLI), mental health, intervention psychosocial/behavioural, parents

## Abstract

**Background and aims:**

A high rate of children in mental health services have poor language skills, but little evidence exists on how mental health support is delivered to and received by children with language needs. This study looked at parental experiences, asking parents of children with speech, language and communication needs (SLCN) about their experiences seeking help for their children's mental health. We were particularly interested on the experiences of parents of children with Developmental Language Disorder (DLD), a specific SLCN that remains relatively unknown to the general public.

**Methods:**

We conducted an online survey of 74 parents of children with speech, language and communication needs (SLCN). Survey respondents included parents of children with a range of difficulties, including DLD, autism, verbal dyspraxia, global intellectual delay, a history of hearing problems, and SLCN without a primary diagnosis. Survey respondents were asked what sources of support they had accessed for their child's mental health and to provide comments on what was good and what was not good about this support. We then conducted 9 semi-structured interviews of parents of children with DLD about their experiences. These were parents of children with DLD aged 7 to 17 years, from across a range of educational settings, and with a range of present mental health concerns.

**Results:**

Content analyses of the survey responses from parents of children with SLCN highlighted three broad factors of importance to parents’ experiences: relational aspects of care, organisational aspects of care, and professionals’ knowledge. Thematic analyses of the interviews of parents of children with DLD identified 5 themes: the effects of language problems on the presentation of distress; the role of the school environment; the role of key professionals; standard approaches to mental health support might not be appropriate; and the role and impact on parents. Parents expressed concerns that their children's mental health problems and need for support would not be recognised, and felt interventions were not accessible, or delivered in a manner that was not comfortable for their children due to high reliance on oral language skills. Some parents were left feeling that there was no provision suitable for their children.

**Conclusions:**

Parents of children with SLCN face barriers accessing support for their children's mental health, including a lack of professional knowledge about their children's language needs. Parents argued that language and communication needs can significantly affect the delivery and success of psychological therapies and interventions. Systematic research is needed to understand how to successfully adapt services to make them accessible to children and young people with language needs, and to ensure that mental health problems are detected in children with language difficulties. Increased knowledge about language disorders such as DLD, and access to speech and language therapy expertise, is needed amongst professionals who work to support children's mental health.

Children and young people with language disorders are more likely than those without language disorders to experience difficulties with their mental health and wellbeing (Botting et al., 2016; Conti-Ramsden & Botting, 2008). These children and young people are over-represented in services for children with emotional or behavioural needs; 80% of children referred solely for emotional behavioural problems have poor language skills (Cohen & Lipsett, 1991; Hollo et al., 2014). Importantly, conditions that affect language development are not confined to childhood, but often persist into adulthood (Clegg et al., 2005), meaning that professionals cannot assume that any additional mental health needs will cease when children's language problems resolve. Given the co-occurrence between language needs and mental health problems, it is pertinent to ask: how can we best support children and young people with language needs to have good mental health? This question will be the focus of the present paper.

To provide suitable context, it is first necessary to describe and define the broad population referred to as having Speech, Language and Communication Needs (SLCN), and also to consider the specific condition Developmental Language Disorder (DLD). SLCN is an umbrella term for a range of difficulties across one or multiple areas of communication including speech, expressive and receptive language, and the social use of language. It is estimated that 10% of children start school with SLCN in the UK, approximately 2–3 children per classroom (Norbury et al., 2016; note that this figure refers to the total number of children described as having a language disorder in this sample. It actually does not include children with severe/complex learning disabilities, who may also have language needs. Thus, as noted by these authors, this percentage is a minimum estimate of the proportion of children in the UK with language needs). Amongst school-aged children (aged 5–16 years) in the UK, 1.6% of all students have an SLCN as their primary SEN (Special Educational Need), and SLCN is the primary area of need for 15.7% of children with an SEN (Lindsay & Strand, 2016). For some children, their SLCN may be explained by the co-occurrence of a known genetic or biomedical condition, and some SLCN may resolve over time and with targeted speech and language therapy input. However, at least 7% of school-aged children have a persistent difficulty learning language with no associated biomedical conditions (Norbury et al., 2016). Children who have a language disorder in the absence of an associated biomedical condition are considered to have Developmental Language Disorder (DLD), previously known as ‘specific language impairment’, amongst other terms (Bishop et al., 2017; note that Norbury et al. (2016) also report the prevalence of DLD under the ICD-10 classification, which has an additional requirement for children to have a nonverbal IQ score of above 85. This criterion was not supported by recent discussions regarding DLD criteria; see Bishop et al., 2017). DLD is equivalent to the DSM-5 condition, “Language Disorder”; however to avoid confusion with the wider group of children who have language needs in the context of other biomedical conditions, we use DLD in this paper to refer to this population.

The prevalence of unidentified language deficits in school children with emotional and behavioural difficulties (EBD) has been previously reported and summarised in meta-analytic papers (Hollo et al., 2014). Understanding the causal mechanisms that link language and mental health problems could provide insights into risk and protective factors for mental health in children and young people with language needs. The exact causal pathways remain unclear but there are a number of likely explanations for the association between language needs and mental health. One possibility is that language and mental health problems are related due to shared genetic effects (Newbury et al., 2019; Toseeb et al., 2022), but this research is still in its infancy. Conti-Ramsden and Botting (2008) found that higher rates of anxiety and depression symptoms in adolescents with DLD did not correlate with language skills, despite the significant group difference between their DLD and control groups. This suggests that language problems indirectly affect mental health through mediating factors. For example, language difficulties may impact children's ability to make and sustain meaningful relationships: by 16 years of age, nearly 40% of adolescents with DLD appear impaired in interactions with peers (Mok et al., 2014), and children with DLD are at higher risk for bullying and victimisation (van den Bedem et al., 2018). These impaired social interactions might then lead to poor mental health in children and young people with language problems. Indeed, language is key in early psychosocial development, such as learning to manage emotions, communicating feelings, and establishing and maintaining relationships (Law et al., 2017) and children with SLCN struggle academically, socially and emotionally (Conti-Ramsden et al., 2009; Durkin & Conti-Ramsden, 2010).

In parallel to research on the causal mechanisms linking language needs and mental health, it is also imperative that we understand what support children with language needs receive for their mental health, and the extent to which support is accessible and effective. Hollo et al. (2014) investigated the prevalence and severity of undiagnosed language deficits in children with emotional behavioural disorders in their meta-analysis. Findings across the participant pool of 1,171 children aged 5–13 years presenting with a formal diagnosis of an emotional behavioural disorders but no previously known language impairment indicated that 4 out of 5 children presented with at least mild language impairment, and 47% of the children showed moderate to severe language problems that had escaped diagnosis. The high prevalence of language needs amongst children with emotional and behavioural disorders suggests that interventions for mental health in children likely need to take into account the co-occurrence of both emotional and language needs, for them to be maximally effective.

Despite clear evidence that the prevalence of language needs in mental health services is elevated, literature on the experience of children and young people with SLCN and conditions like DLD in mental health services is lacking. Some insight can be drawn from examining the literature on other neurodevelopmental conditions. Studies on the experiences of autistic children and young people of CAMHS (Child and Adolescent Mental Health Services) suggest mainstream interventions that are unadjusted to take into account a young person's autism usually failed to improve the mental health of children diagnosed with autism, or in some cases worsened their mental health (Read & Schofield, 2010). Parents also reported that their children's mental health difficulties were dismissed and labelled as being a feature of having autism, rather than a condition in its own right (Read & Schofield, 2010). Similar results have been found for autistic adults’ experiences. Autistic adults found it hard to access treatment and support for mental health, suicidality, and self-harm, and faced a lack of understanding and knowledge about mental health and autism: this poor knowledge about autism was seen to impact negatively on people's treatment experiences (Camm-Crosbie et al., 2019). A recent meta-analysis of 12 studies found that psychological therapies could yield positive effects for autistic people, but yet again a common barrier was a lack of therapist knowledge or expertise in autism, and therapists’ inability (or, as perceived by some participants, unwillingness) to tailor approaches to support the needs of autistic people (Adams & Young, 2021). The research in autism and mental health services suggests that a lack of professional knowledge and a lack of adjustment for communication needs could impact care for affected children and young people.

This has implications for those with language difficulties, who similar to children and young people with autism may need professionals to have specialist knowledge, and adjust their support. However, relative to autism, DLD, and childhood language problems generally, are less well known by the public (McGregor, 2020; Thordardottir & Topbaş, 2021), and DLD remains an under-researched condition, relative to other neurodevelopmental conditions (Bishop & Morty, 2010; McGregor, 2020). Many educational practitioners lack clarity on what constitutes an SLCN, and are unaware of terms such as DLD (Gallagher et al., 2019a). Part of this poor awareness is due to a wide range of different terms and labels being used historically, often to refer to the same or overlapping populations of children: to resolve this, researchers and practitioners have advocated the use of the term DLD to refer to causes of language disorder not associated with another biomedical disorder (Bishop et al., 2017). Speech and Language Therapists (SLTs), teachers, parents and children with DLD when asked to describe optimal goals and interventions for children with DLD lack commonality, with notable differences between the professionals on goals (Gallagher et al., 2019b). This may extend to mental health professionals, though there has been limited systematic study of the knowledge base of these groups concerning DLD and SLCN. However, it might be expected that children with SLCN have at least similar, if not poorer, experiences to children and young people with autism.

The perspectives of parents are particularly important and useful in the context of this topic. Discussing mental health difficulties, and the experiences of mental health support, with children with SLCN themselves is important but challenging, given individuals’ language and communication needs. Parents can therefore provide a useful perspective, providing a framework for future research accessing the views of the children and young people themselves.

The present study aimed to investigate the experiences of parents of children with SLCN, and in particular DLD, when accessing and receiving mental health support. We aimed to map out key barriers to getting timely support, and highlight facilitatory factors and practises that are perceived to work well by parents.

## Method

### Design

**Figure 1.**
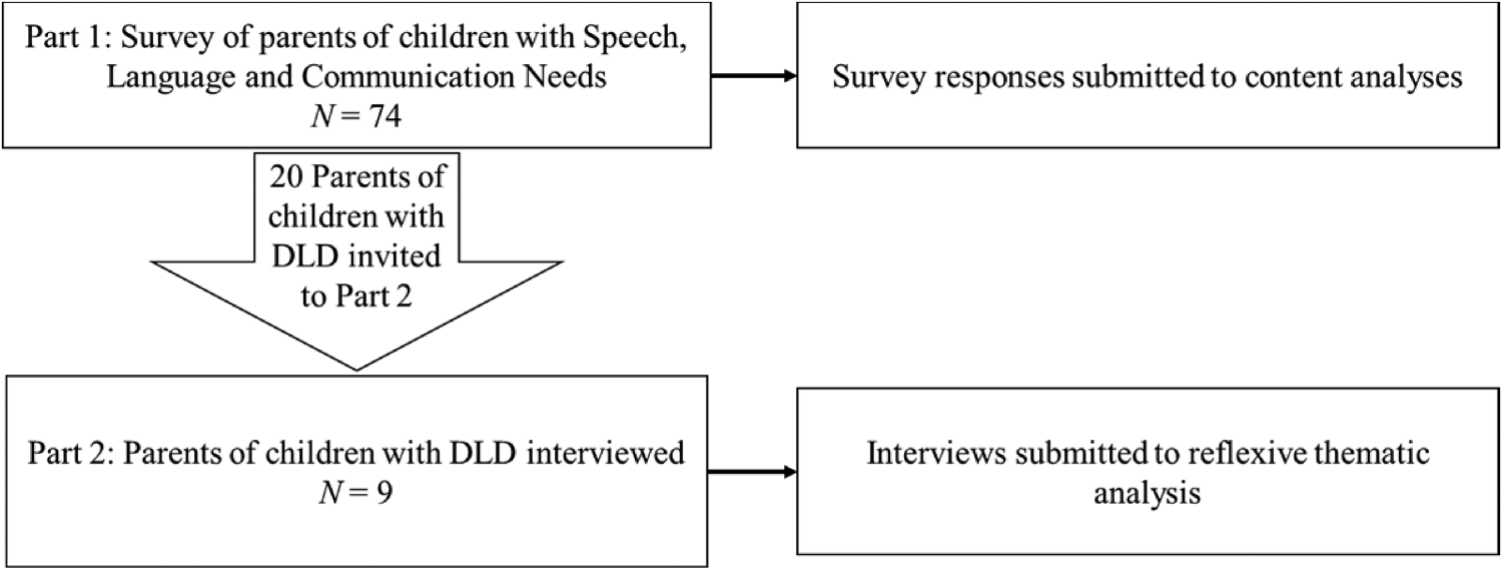
A summary of the structure of the overarching project and recruitment.

Our study was designed with two stages. The first part was a survey for parents of children with SLCN about their concerns about their children's mental health and their experiences getting support. The second part consisted of online interviews with selected participants from the survey respondents, who were parents of children with DLD. We opted for this approach as we wanted to a) understand the difficulties that children with DLD and their families face, in the context of other SLCN and b) we were concerned that the relative lack of awareness of DLD might impact recruitment for stage two. For clarity, the procedure and participants that were included in the survey and interviews are described separately. Figure 1 provides a visual summary of the two parts of the project.

### Part 1: survey of parents of children with SLCN

#### Survey participants

In part one of the study, parents of children with SLCN were recruited from SLTs listed on the Association of Speech and Therapists in Independent Practise website, charities, schools and DLD Engage Platform (see https://www.engage-dld.com/). There were 74 survey responses that were recorded and could be used in the analyses: participants who only left demographic information but no responses to any questions about the care they received were not included in the analyses. Table 1 summarises the participant characteristics of the survey participants. The survey sample included parents of children with DLD, autism, a history of hearing problems, verbal dyspraxia, language needs in the context of global intellectual disabilities, and children who had speech and language concerns but no formal diagnosis.

**Table 1.** Characteristics of participants for part 1 survey (N = 74).

Age of child	M = 10.26 (SD = 4.09)
Gender of child	Female = 22 (29.73%), Male = 52 (70.27%)
Diagnosis (SLCN)	DLD = 40 (54.05%)
	ASD = 18 (24.32%)
	Global Intellectual Disability = 4 (4.05%)
	Verbal Dyspraxia = 3 (4.05%)
	History of hearing impairment = 8 (10.81%)
	None of the above = 16 (21.62%)
Mental health diagnosis	Anxiety = 6 (10.81%)
	Depression = 2 (2.70%)
	Other = 6 (8.12%)
Survey respondent's relationship to child	Mother = 69 (93.24%)
	Father = 3 (94.05%)
	Legal Guardian = 2 (2.70%)
Education level of survey respondent	Doctorate or Professional Degree = 7 (9.46%)
	Master's Degree = 8 (10.81%)
	Bachelor's Degree = 20 (27.03%)
	Completed Sixth Form = 19 (25.68%)
	Completed Secondary School = 17 (22.97%)
	Prefer not to say = 3 (4.05%)

#### Survey procedure

Parents were asked to complete a short online questionnaire. It included questions about demographic information, including details of children's SLCN diagnoses and mental health. The survey then went on to ask about mental health support, specifically: where they went for support (listed options included their child's school, their general practitioner (GP), an SLT, a counsellor/psychologist, or the option to specify “other” and give more details); their opinions of the support given; how satisfied they were with the support; what (if anything) prevented them from seeking support; if any provisions were put in place to account for their child's language difficulties; and any changes they would like to see to the support for mental health for children with SLCN. A copy of the questions used in the survey can be found online: https://osf.io/uwvz2/. As a thank you for their time, parents were also given a choice of three charities for the researchers to donate £2. Parents had the option to leave their email if they wished to be contacted about part two of the study.

### Part 2: interviews of parents of children with DLD

#### Interview participants

Participants for part two of the study were selected from those who had agreed to be contacted after completing the part one survey. Parents were invited on the basis that they had a child with a diagnosis of DLD, and that they had had some concerns in the past or at present about their child's mental health (parents who expressing having never had concerns were not invited). They were not invited on the basis of their feedback about any of the sources of support they had accessed in the survey. Two parents in the survey had children with dual diagnoses of DLD and Autism Spectrum Disorder (ASD), or DLD and Foetal Alcohol Syndrome. It was decided that these children's experiences would likely be very different from those without diagnoses in addition to their DLD, and for the sake of keeping some homogeneity within the interview participants these two parents were not invited to the interview stage. Twenty participants were contacted regarding being interviewed, and nine agreed and subsequently completed the interviews, all mothers. Children's ages ranged from 7 to 17 years. Four parents had children in primary school, 4 in secondary school and 1 in college. Table 2 summarises the characteristics of the interview participants.

**Table 2.** Characteristics of participants for part 2 interview (N = 9).

Age of child	M = 12.00 (7–17)
Gender of child	Female = 2, Male = 7
Education level of parent	Bachelor's Degree = 2
	Completed Secondary School = 2
	Master's Degree = 3
	Doctorate or Professional Degree = 2

In order to help characterise their children's current communication and mental health functioning, parents also completed two questionnaires. Table 3 summarises the scores obtained on these measures. Note that these were not outcome variables, but rather simply used to describe the current sample.

**Table 3.** CCC and SDQ scores for interview sample.

Children's Communication Checklist Scale Scores		Strengths and Difficulties Scale Scores	
	Mean (minimum-maximum)		Mean (minimum-maximum)
Speech scale	28.78 (22–34)	Emotion	4.11 (0–8)
Syntax scale	30.67 (28–32)	Conduct	1.33 (0–4)
Social scale	29.75 (26–33)	Peer	2.44 (0–7)
Interests scale	29.75 (27–34)	Hyperactivity	4.11 (2–6)
Pragmatic composite score	133.44 (114–147)	Pro social	3.56 (2–5)
		Impact	3.89 (1–10)
		Total*	12.00 (6–20)

*Total difficulties score is the sum Emotion, Conduct, Peer and Hyperactivity scales.

**Parent-report child's communication difficulties.** Parents completed the Children's Communication Checklist (CCC; Bishop, 1998), which is an 70 item questionnaire, consisting of 9 subscales, each measuring a different aspect of language and or communication competence. The subscales are speech, syntax, inappropriate initiation, coherence, stereotyped conversation, use of context, rapport, social skills and interests. Parents responded on a 3-point scale (0 = does not apply, 1 = applies somewhat, 2 = definitely applies) to indicate the extent to which children showed particular behaviours (e.g. “People can understand virtually everything he/she says”, or “Their speech is clearly articulated and fluent”). The CCC was developed to capture pragmatic language problems in particular, reflected in the pragmatic language composite, which combines the scores for inappropriate initiation, coherence, stereotyped conversation, use of context and rapport. Scores of below 140 have been recommended to indicate pragmatic language problems (note that for this version of the CCC, higher scores on the questionnaire indicate better communication skills). Six of the children of the parents interviewed scored below this threshold.

**Parent-report child's mental health.** The parent-report Strengths and Difficulties Questionnaire (SDQ; Goodman, 1997) was used to measure children's mental health difficulties. The SDQ is a valid screening instrument for common mental health difficulties in samples of neurodiverse children (Bryant et al., 2020). The questionnaire consists of 25 items, which can be divided into five subscales. These are emotional difficulties, conduct problems, peer problems, hyperactivity, and prosocial behaviour. The impact supplement (in which parents indicate the extent to which their children's difficulties are impacting them in different aspects of life) was also completed. Parents responded to each of the questions on a three-point scale (0 = not true, 1 = somewhat true, 2 = certainly true). Total SDQ scores for the current sample ranged between 6 and 20. Total SDQ scores and impact scores can be used to assign children to one of four groups in terms of their presence of problems (these bands are: close to average, slightly raised, high and very high). Using the four-band thresholds for the SDQ, five children's current total SDQ scores would be considered close to average, two to have slightly raised behavioural problems, one would be considered to have high scores, and one would be considered to have very high scores. Examining the impact scores, seven children would be considered to have problems that had a very high impact, one to be considered high impact and one a slight impact.

### Interview procedure

Parents participated in a semi-structured interview over Zoom. Our interview topic-guide can be seen on the Open Science Framework page for this project. Before the interviews, parents were also asked to complete an additional consent form for audio video recording. Interview recordings were transcribed to be fully anonymised with names, schools and places removed.

### Ethical considerations

Both the survey and interview parts of the study received ethical review by the University of York Department of Psychology ethics committee (Reference: 804). All participants gave informed consent, and additional consent was required prior to the recording of the interviews.

## Results

### Analytical approach

For our survey, quantitative statistics are used to summarise parents’ ratings of satisfaction for each of the sources of support they accessed. Survey responses to open text questions were analysed using content analyses. Categories were developed for each source of support. We then reviewed the overlap and similarities between categories across sources. This led to the development of three supra-categories, which summarise commonalities in what it important to parents about their care, whichever source of support they access. The anonymised survey results are available to view on the Open Science Framework: https://osf.io/uwvz2/.

Interview transcripts were coded using an inductive approach, generating codes and themes from our data. Codes were grouped and developed into candidate themes, according to the reflexive thematic analysis approach (Braun & Clarke, 2006). A semantic and critical realist approach was taken. Critical realist approaches separate structures and mechanisms (the real) that generate events (the actual), which may then be experienced and perceived (the empirical) (Willig, 2013). This allows the experiences of individuals, and their reports, meanings and reality to be fully recognised.

The results of the survey data are presented first, followed by thematic analysis of the interview data.

### Mental health concerns of parents of children with SLCN

Parents were asked about how concerned they were at present about their child's mental health, and how concerned they had been in the past (See Table 4). 44.59% of parents reported having been very concerned in the past, and 60.81% remained quite concerned. In addition, we asked whether their child had received any therapy, counselling or an intervention aimed at their mental health: of those who responded (N = 72), 63.89% told us they had not received any interventions for their children's mental health.

**Table 4.** Mental health concerns of parents and schools.

	How concerned about your child's mental health have you been in the past?	How concerned are you about your child's mental health at present?
Not very concerned	13.51%	24.32%
Quite concerned	41.89%	60.81%
Very concerned	44.59%	14.86%

### Satisfaction and key factors across different sources of support

**Figure 2.**
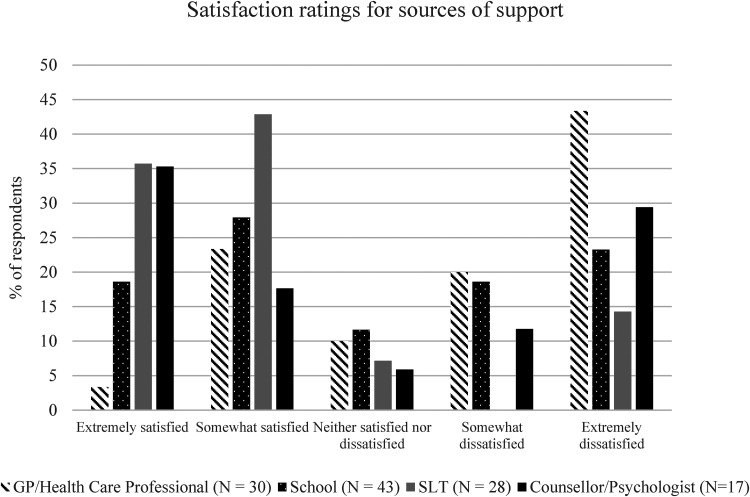
Distributions of satisfaction ratings for 4 main sources of support for mental health.

Parents were asked if they had accessed support from their child's school, their GP, their SLT, or counsellors and psychologists. For sources parents had accessed, they were asked to rate their satisfaction with their support. These scores are summarised in Figure 2. Parents were able to note other sources of support that they had accessed: 5 parents listed CAMHS but did not rate this source of support or leave comments that could be integrated into the content analyses. “Other” sources of support also included parents who had visited a massage or acupuncturist (*N* = 1), or support services in their local area (*N* = 1).

**Figure 3.**
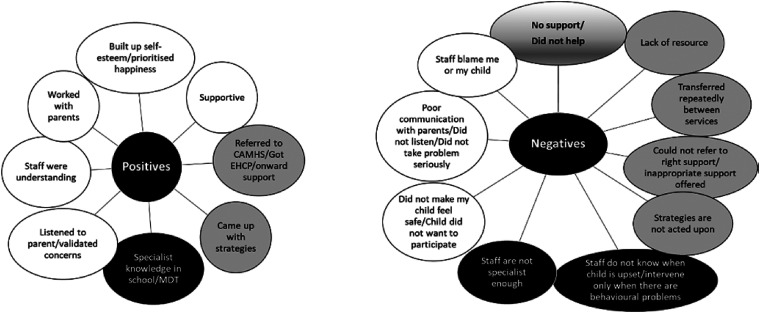
Categories of responses of parents about support from their children's school. 31 parents gave positive comments, and 27 gave negative comments. Black categories are considered issues of professional knowledge; white categories are considered issues of relational aspects of care; grey categories are considered issues of organisational aspects of care. Some categories are considered a mixture (these categories have a mixture of colours).

**Figure 4.**
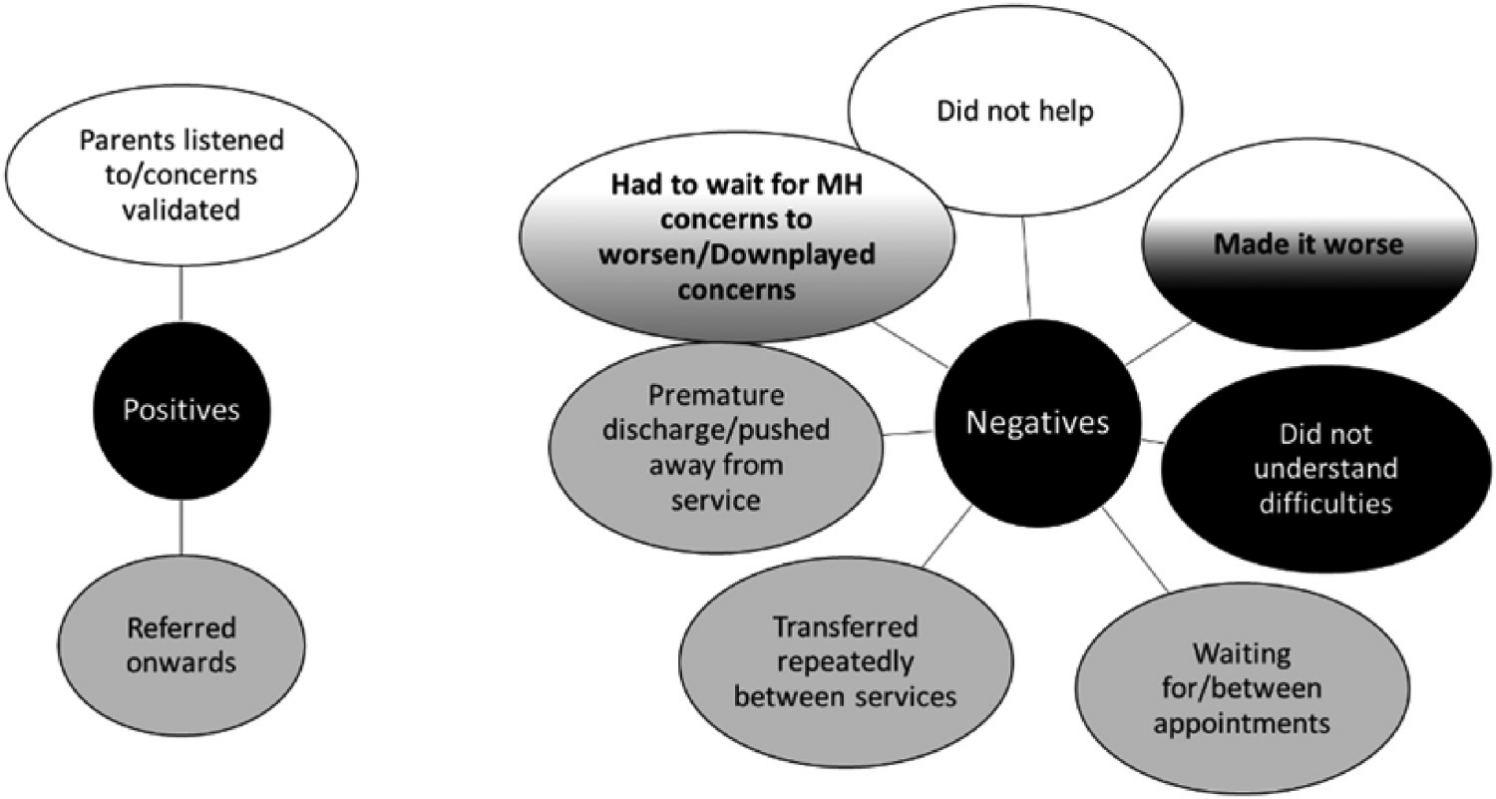
Categories of responses of parents about support from their children's GP, or health care professionals. 15 parents gave positive comments, and 25 gave negative comments. Black categories are considered issues of professional knowledge; white categories are considered issues of relational aspects of care; grey categories are considered issues of organisational aspects of care. Some categories are considered a mixture (these categories have a mixture of colours).

**Figure 5.**
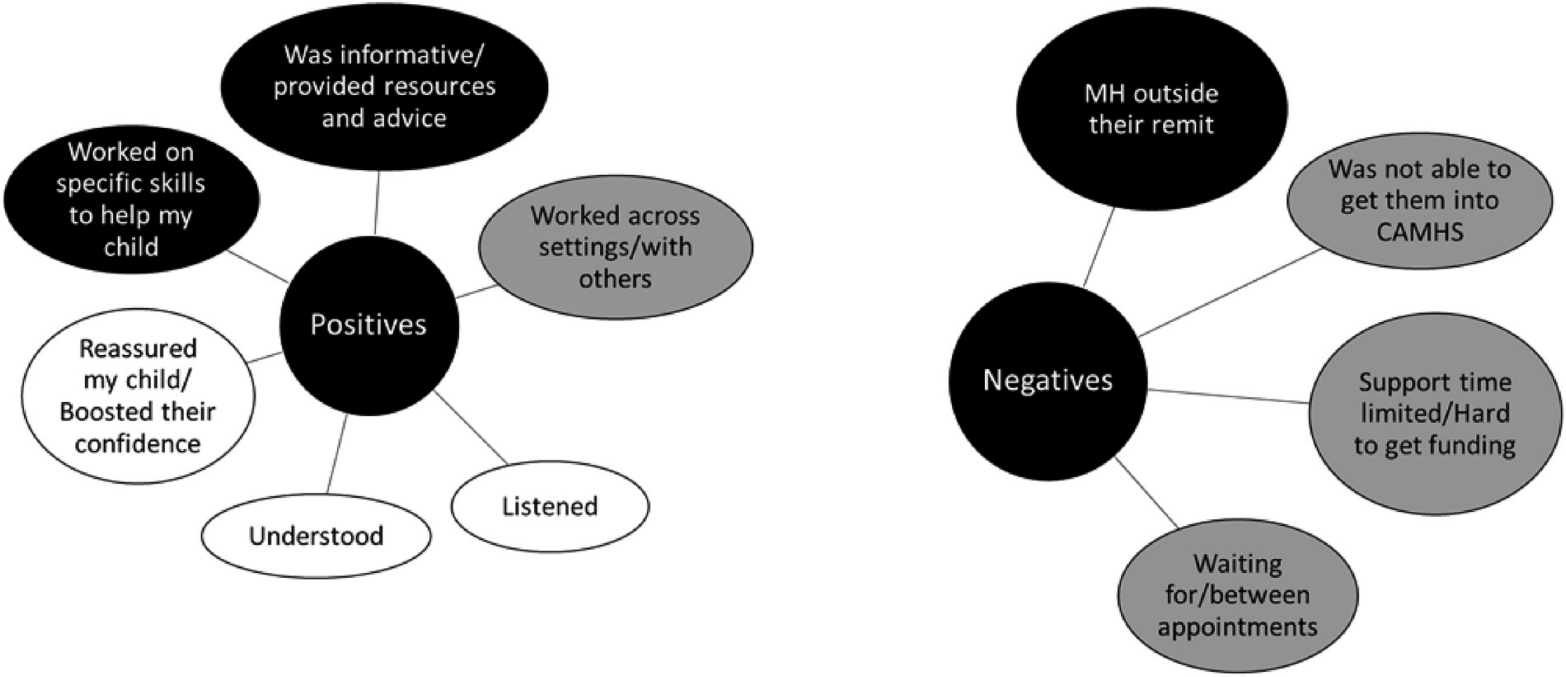
Categories of responses of parents about support from their children's SLT. 20 parents gave positive comments, and 19 gave negative comments. Black categories are considered issues of professional knowledge; white categories are considered issues of relational aspects of care; grey categories are considered issues of organisational aspects of care. Some categories are considered a mixture (these categories have a mixture of colours).

**Figure 6.**
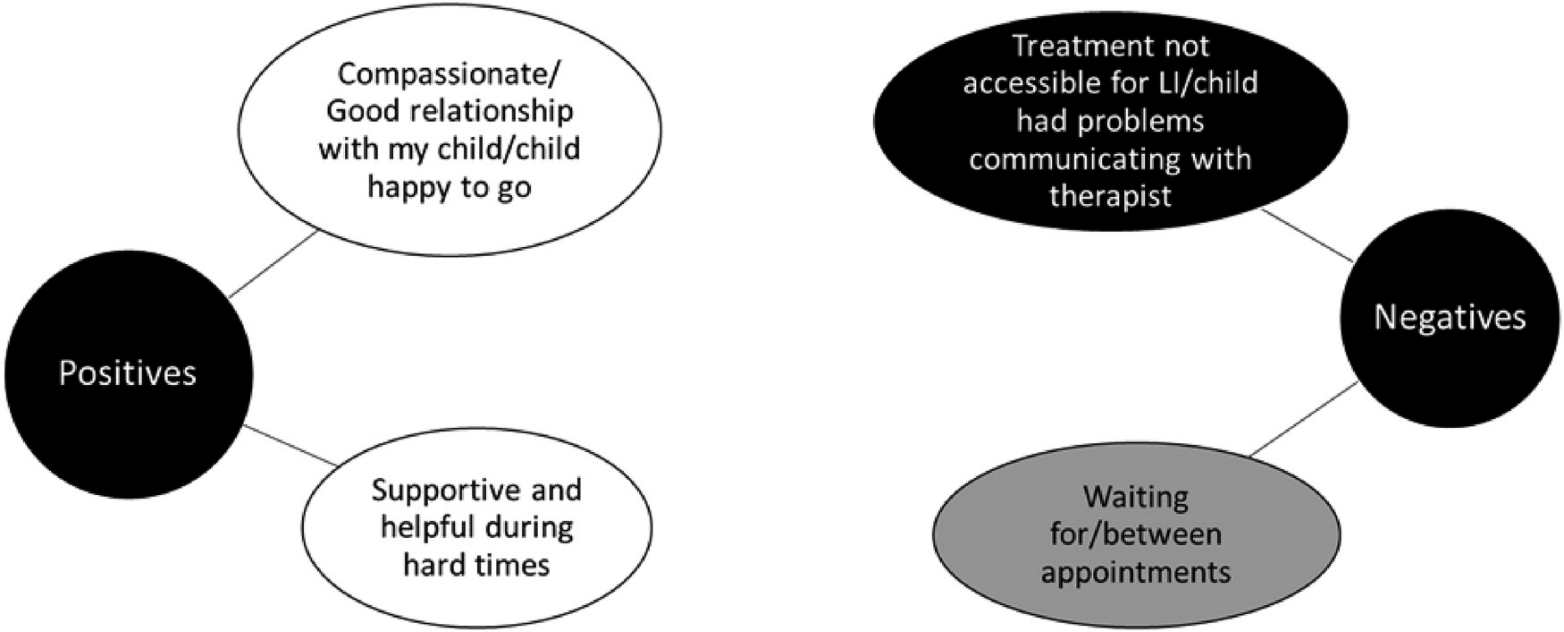
Categories of responses of parents about support from their children's psychologist or counsellor. 14 parents gave positive comments, and 13 gave negative comments. Black categories are considered issues of professional knowledge; white categories are considered issues of relational aspects of care; grey categories are considered issues of organisational aspects of care. Some categories are considered a mixture (these categories have a mixture of colours).

Examining parents’ positive and negative comments about the care they received across all sources accessed, responses were reviewed and patterns emerged to support three key factors. These included the relational aspects of care (which appear in white, across Figures 3 to 6), organisational aspects of care (shown in Figures 3 to 6 in grey) and professional knowledge (shown in Figures 3 to 6 in black).

Relational aspects of care refers to comments highlighting that parents wanted to be listened to, taken seriously, and not blamed for their child's difficulties. This factor also captures comments about how their child was treated: that their child was helped to feel safe, and that professionals worked to help their child build their confidence, and have good self-esteem.

Organisational aspects of care reflects the structure of care, and resourcing. These comments reflect that parents wanted timely referrals, particularly to CAMHS, and support for getting an EHCP (Education Health Care Plan; these plans are used in the UK to identify educational, health and social needs and to set out the additional support children require to meet those needs, where school's usual special educational needs provision is not enough to support a child). Waiting times were mentioned across multiple sources of support, as was experiences of transferred or directed repeatedly between services. Where parents have stated that there was no help available to them, this could be considered both a relational comment, but also an organisational one: some comments reflected a feeling that there was no service appropriate for their child's needs.

The final factor across sources of support was professional knowledge. Parents wanted professionals to be suitably versed in the child's needs, or access professionals who were. Their comments also reflected concerns that staff were not suitably trained to detect mental health problems in their children, or modify their treatment approaches to support them.

To provide a more in-depth summary of parents’ experiences with each of the four main sources of support investigated, we summarise parents’ qualitative comments for each source of support below.

#### Support from school for children with SLCN

The categories developed for the positives and negatives about the support parents received from their child's school are summarised in Figure 3. In positive comments, staff were described as being supportive and understanding, and parents appreciated when staff listened to their concerns and took them seriously, and when they worked with the family to help support their child. Some parents commented that their school helped their child to improve their confidence, prioritising their child's happiness over academic achievements. Parents liked the specialist knowledge some of their school staff had, and appreciated when staff worked with other professional groups to get specialist input to help their child. Staff developed strategies to help their child, and parents also commented on the role that schools had in getting access to other sources of support such as CAMHS, or supporting them through the formal process of getting an EHCP.

Considering the negatives reported by some parents, some complained that the school staff did not communicate with parents, did not listen, or did not take their concerns seriously. Others were disappointed about the lack of specialist knowledge available in their school. For some parents, schools were not able to support parents to access onward support. Some felt that school staff had negative perceptions of them or their child, and blamed them for their difficulties. They felt repeatedly transferred and directed back and forth between services, or complained that there was a lack of resource (this was usually described as staff not having enough time with their child). For some, although strategies might have been developed, these were not acted upon. Some parents were also concerned that staff were not able to spot when their child was upset, or that only children who were disruptive would receive any help for their mental health.

#### Support from GPs and healthcare professionals for children with SLCN

It should be noted that while our survey asked about GPs specifically, it was clear from comments that many parents were not just commenting on their interactions with their GP, but other healthcare professionals in general, including paediatricians and CAMHS professionals. This source of support received the worst ratings in terms of satisfaction, but it is unclear to what extent this was necessarily due to the support of GPs themselves, or the services that GPs referred them onto.

Two dominant categories of response were developed to summarise the positive comments about GPs and healthcare professionals: i) that many parents felt listened to and had their concerns validated ii) many parents felt they were referred to appropriate support (usually CAMHS). Together, it appears that parents’ main expectations for their primary health care professionals is that they listen, believe parents, and make a timely and effective referral to appropriate specialist support.

However, more categories of negatives emerged within the responses. Some of these were organisational in nature: parents reported feeling prematurely discharged from services, or some parents said they felt like the clinician thought that they or their children were unengaged in services. Others reported being transferred and referred repeatedly across services, or experiencing lengthy waits between appointments. For some, they reported their children's mental health had to deteriorate further before help was offered. Parents complained that they did not receive help from their health care professionals, or even that some interventions made their child's mental health worse. Finally, some parents reported their health care professionals did not understand their child's difficulties (there were examples pertaining both to their mental health difficulties and their language and communication difficulties).

#### Support from speech and language therapists for children with SLCN

Speech language therapists were the group that received the highest satisfaction ratings. Parents reported that their speech and language therapist was informative, able to provide resources and advice, and worked on specific skills with their child (for example, social skills). Parents were pleased to see SLTs working with other professionals around their child, working with their children's schoolteachers, and across different settings. Parents also commented that their SLT listened to them, understood their difficulties, and provided reassurance to their chid, boosting their confidence.

The negative comments relating to SLT care were largely in relation to the organisational aspects, particularly resourcing of care. Parents commented on the difficulty getting into this sort of support, that it had been hard to get funding, and that it was funded only for a limited time (and indeed, several parents reported they had paid for private speech and language input). There were also lengthy waits between sessions. Also noted were parents commenting on the need for specialist support for their children's mental health, but SLTs were not always able to help them gain access to CAMHS, with mental health per se being considered outside of their SLT's remit.

#### Support from counsellors and psychologists for children with SLCN

Some comments about experiences in CAMHS were described when parents were asked about their care from their GP: thus, it is not clear whether the feedback on these sources should be considered as completely separate from their comments on their interactions with GPs and their healthcare professionals. Nonetheless, we present the content analyses for the comments left when parents were asked specifically about their experiences with counsellors and psychologists.

Positive categories highlighted the relational aspects of care parents received: positive comments including feeling that their psychologist or counsellor was compassionate and had a good relationship with their child, which helped the children engage with the service. Parents also noted they were supportive, and provided emotional support during difficult periods.

However, several parents also argued that their psychologist or counsellor had not made treatments and approaches accessible to their child: “*talking therapy*” was mentioned by some parents as a non-accessible treatment approach for children with language needs. The other recurrent negative theme was the time waiting to be seen by a service and length of time experienced waiting between appointments.

#### Thematic analysis of interview data

A thematic analysis was conducted on interview transcriptions, from which 5 core themes were developed. These themes spanned the presentation of mental health needs and distress through to impacts upon treatment and support, and touched on the roles of the school environment, professionals, and parents themselves. In addition to the quotes used in the main text, our supplementary materials include Tables with further quotes, listed under our themes.

#### Language needs affect detection and presentation of distress

Reports from parents helped suggest the way in which children with DLD may present mental health problems. Parents described their children showing difficulties communicating their distress, and struggling to report when negative events, such as bullying, had occurred. In some cases, children were extremely distressed, and even later admitted to feeling suicidal, having not had the ability to express their feelings at the time: not realising the extent of their child's upset was clearly distressing for parents as well. Some parents expressed concern that these issues could leave their child vulnerable even into adulthood.

There was variability in the extent to which children showed externalising versus internalising behaviours, and these issues had consequences for how children were viewed and recognised. While some parents felt staff had negative perceptions of their child due to their externalising behaviours, others expressed concern that support tended to go to children who were showing more overt behavioural problems, meaning their children with more internalising concerns were left out of needed support.

“…at one point I felt that he was very distressed, and he should’ve been offered that [support] earlier. And I felt that because he wasn't showing it in a way, the behavioural way, that some of the other children were getting the therapy, that's why he wasn't being offered it. So that's obviously always a worry because my son is very self-contained…If you don't show it behaviourally, that's what systems respond to and you know services respond to, so that's always a worry for me.” – Parent 1

There were other characteristics of distress that recurred in our sample. Several parents reported children showing somatic symptoms, such as sleeping and eating problems, which reflected children's stress and anxieties. Several parents also reported that their children were very nervous outside the home or away from their caregiver, particularly in new environments. Some children had developed a strong sense of safety within their home with their primary caregiver, which appeared as a clinginess to their parent. Parents drew links between their child's fears leaving the home, or being apart from them, and their children's language needs: one parent argued that their child's experience in the outside world would be like being dropped into a country where you do not know the language. Outside the family home, the environment could be unpredictable, and children might feel unable to ask for help or reassurance from those who did not understand them.

“…he was quite happy in his own home, in his own bubble, but as soon as I had to get him out the front door, particularly going to school, then it just went all horribly wrong, so yeah and it was down to I think the communication and the lack of understanding of people.” – Parent 6

#### Traditional methods may not be appropriate for children with DLD

From the reports of our interviewed parents, it appears some felt that traditional methods of supporting children with their mental health and wellbeing, such as *talking therapy*, may not always be the most suitable sources of intervention. A number of parents had ideas about alternative sources of provision or tools that could help.

Parents’ reports appeared to suggest that traditional face-to-face talking therapy with their children was not effective. It was mentioned that interventions not supported through visual aids were uncomfortable and unsuitable for a child with DLD, especially given that difficult, emotional conversations may be anxiety-provoking for children who find talking and conversations hard. Other parents mentioned that they felt play therapy would be something that would work for their child better, if activities were pitched at the right age. Similarly, several parents mentioned activity-focused interventions, or interventions that did not simply involve a therapist and child talking to each other; these were considered more likely to be successful. This appeared to give children some to focus on and build rapport around with the therapist.

“He built a bow and arrow in the wood, went fishing, so that it wasn't sit down and talk, because I knew that wouldn't work, and I communicated quite clearly about how it might become difficult for CHILD to engage with words. So he [the therapist] worked with CHILD in a very gentle way in different environments, and so language got taught then for expressing…it was a gentle approach” – Parent 8

The value of additional visual supports or using technology to support children to communicate their mental states was also noted by parents. One parent described using an app with a scale for their child to rate their mood, which was more approachable for the child than simply holding face to face conversations. This use of technology allowed the children to more successfully signal to key staff when they were struggling with their emotional wellbeing.

In addition to the setting and delivery of traditional treatments and interventions, the content was also not always accessible for children with DLD. One parent attended a course for parents on supporting and managing their child's anxiety, a course run by CAMHS. Despite attending the course for a younger chronological age group than their child, the language and techniques provided were still not appropriate for their child with DLD. As a result, the parent had to simplify and modify the material themselves to make it appropriate to use at home.

#### Role of the school environment

Children in our sample were in a range of educational settings, including primary, secondary and further education stages, and including mainstream and specialist environments. The school environment appeared pivotal to the experiences of many of the families. Some parents were very happy with the support they had received from their schools. However, in some instances parents felt that their school appeared to exacerbate their children's mental health problems. The impact of specific interactions with school staff is covered in the theme of “the role of key professionals”, but here we reflect on key aspects of children's school environments, and their experience throughout their school journeys.

Schools that worked with and listened to concerns of parents were predominately described as nurturing. Teaching staff were accommodating and understanding, and were reported to make efforts to ensure the child felt safe in the school environment. It was not always that the child was in a specialist school or part of a language unit when parents viewed their child's school as nurturing, but rather that professionals were caring and supportive to the child. For example, one parent discussed the extensive measures a secondary school put in place prior to the transition to secondary school (such as day trips away from the school grounds). Another parent reported that her child's school's resource base put on a birthday party for a member of staff, as staff realised that almost none of the children with SLCN in that class had ever been invited to a birthday party. Another parent said how she appreciated that school staff listened to her requests about managing her son's eating at school, not pressing him to eat more, as this could trigger his anxiety.

“They were so nurturing and kind, to me and to him, so they I think made him feel safe – as safe as they could in a big busy school. And things like his eating does play out in school…. And they kind of just made it okay for me to put whatever it took for him to eat in his lunchbox – stuff that I was like ‘I can't put that in because what will they think’, and they were like put it in, we don't care, we just want to see him eating.”– Parent 3

Others’ experiences were less positive. One parent reported that her daughter's teacher had deliberately taken away many of the strategies her daughter had used to cope in the classroom: while this likely highlighted her daughter's previously unrecognised communication needs, the experience impacted her self-esteem. Transitions also came up frequently in our interviews, and in one example offered a clear example of how much the young person's previous school environment had been affecting his mental health. The parent had expected things would be even worse at secondary school, but instead the change of environment seemed to offer a chance to reset this child's relationship to school. The attention and support received from the new teachers before during and after the transition meant that this child began to thrive at their new school. This child compared negative or stressful life events to their time at their primary school.

“Even now he sort of reflects back on to his time at PRIMARY SCHOOL and it's almost like post-traumatic stress … at the appointment, he was reflecting back on his time at PRIMARY SCHOOL and he was still very traumatized by it and everything was measured against what had happened at PRIMARY SCHOOL. So he would relate everything back to PRIMARY SCHOOL – anything bad he was like, oh, this is like being taught by his old teacher, or my time that I got cross in the trees…” – Parent 6

One aspect of the school experience that was noted by parents was the peer relationships children formed, or did not form. It was often the case that children with DLD reported feeling lonely in school. Some parents wished that schools had taken a more active role in supporting their child to make friends. For children who had trouble attending school, or who when in school were taken out of class and away from their friends, infrequent contact with peers, or doing different work to their classmates, compounded their isolation. This was the case especially as their friends became older and it became harder for them to keep up with the social group. For some parents, their children's loneliness was their dominant concern. For others, their children's social relationships provided a protective factor.

“…it [the parents’ concerns about their child's mental health] all stems from the same thing which is fundamentally his ability to build relationships with other children, with adults, and to not just make a sort of link to people, but to sort of develop that into a satisfying, lasting friendship, whatever it may be. I think that [his ability to form these relationships] has obviously been hampered by his speech and language development.” – Parent 2

#### Role of key professionals

Children and families in our sample interacted with a wide variety of professionals, including SLTs, SENCOs, mental health practitioners, educational psychologists and psychiatrists. Parents often had the task of connecting up these disparate groups. While some professionals proved to be useful sources of support and help, others were depicted as a gatekeeper for parents trying to access support. This theme considers what professionals did (or did not do) that helped or hindered supporting children with DLD.

Past experiences of having their concerns downplayed by professionals had clearly left strong impressions on the parents. Frequently parents described their impressions of talking to school professionals as being made to feel they were being overly concerned, taking up staff time or, as some parents put it, “making a fuss”. When parents were asked what advice they would give to parents in a similar situation to themselves, parents used phrases such as “keep pushing” or “keep fighting”. Many parents reflected on their experiences of getting their children's language needs recognised, when their concerns were often not validated by professionals. This in some cases led to a breakdown of trust between parents and the school. One example of this was a case where a parent, who themselves had a professional background in education, re-applied the same assessment at home (borrowed from their place of work) because they felt so doubtful that the school staff had conducted in properly.

Possibly these experiences when getting their child's DLD recognised and diagnosed coloured parents’ expectations of mental health support. There was a sense of wariness when approaching professionals for support: parents knew they needed to argue the case for their child's support to professionals, and several parents knew through friends that approaching mental health services was unlikely to be fruitful if their child's difficulties were not severe enough (and indeed, some parents had faced problems getting referrals for support even when their child's mental health was at crisis point). Some parents discussed how they felt able to discuss their child's mental health with the speech and language therapist; however, when they did this, the speech and language therapist was not able to give advice for mental health.

In cases where parents did pursue support from mental health services, many families were often met with a lack of awareness and knowledge of DLD. This meant parents had to explain what DLD was and their child's needs repeatedly, which many described exhausting and frustrating. In some cases, parents reported that the mental health workers did not seem to understand DLD, and this impacted negatively upon the therapeutic process. Parents shared there were then problems with misunderstandings and building trust and rapport between themselves and the professionals.

“I remember one session with CAMHS… they said how did you get here and he said by car – he was answering so literally that they kind of felt that he was kind of taking the mick, and he wasn't, and I was really angry with the therapist because he kind of was like quite pissed off with my son. Whereas I was like, you just don't get it. And that was my first experience – that was many years ago, and that was when I realised that there was so little understanding of language disorders, really”. – Parent 8

Of course, input from SLTs could help support other disciplines to work with children with DLD, but parents reported that professionals did not appear to work together in a manner that would best support their child. One parent told us that their child had been part of a group intervention at school, delivered by a clinical psychologist, and it was not until near the end of this intervention that the psychologist learned of their child's DLD diagnosis (when the parent themselves told them about it). In cases where parents linked their speech language therapists with other professionals working with their children, parents expressed their frustration at having to do this rather than it being done by the professionals themselves.

“Because they just don't ever work together, they never work together. I literally years ago told one of the CAMHS workers to look out of his window to another window and wave to the speech and language therapist that's sitting there because if you actually – you know, you park in the same car park and you are in the same building and you could just literally talk in the car park about my children”– Parent 8

#### Role of and impact on parents

Parents took on many roles to support their children, and having a child with DLD impacted many aspects of multiple parents’ lives including finances, their social and professional lives, and their own wellbeing. This theme distils some of the recurrent roles and impacts we heard in our interviews.

Parents took on the role of acting as a translator for their child when interacting with peers and professionals, translating what their child was saying and how they were feeling. With friends and family, they did this in a way that was not obvious to their child, to protect them from the negative feelings of not being understood. However, this fed into feelings of children being very dependent on their parents, and could underlie some of the anxiety children seemed to show about being away from their home.

“Yes, I’m very much a translator - her and I are almost joined at the hip, you know, and that's difficult because if you took me away then you show her vulnerabilities very much, unless there's other understanding adults.” – Parent 5

Parents also had a central role as the advocate. Parents frequently described their quest for getting services and support as a fight or a battle. In some instances, parents reported feeling like they were educating professionals about DLD. Notably, as many of our sample of parents were themselves from professional backgrounds including health care and education, we asked whether they felt their role might have affected their experiences: all agreed that their professional backgrounds had provided an advantage, allowing them to have the right terminology, or insight into systems, that helped them advocate effectively.

Parents also had a clear role in linking up different teams and services. Parents were liaising with multiple services, in health care and in education, and described experiences of being repeatedly transferred and referred between services, which led to delays in their children's support.

“I’ve had to make it easy for them, I’ve had to say look, I have a speech and language therapist that's been working with him for the past five years, I can give you her number and she will come into college and work with you. So I’ve basically had to be that link”. – Parent 9

Their roles as translator, advocate and central liaison appeared to be exhausting for parents. Many parents reported that their experiences going back and forth being many different services was frustrating, coupled with the feeling that there were not the appropriate services set up for their children. Some parents reflected on the suspicion that this would be a role they would need to fill it for the rest of their life. Some parents reported quitting their jobs during the tribunal process due to stress. It was clearthat many parents felt like they had no appropriate service or support for their child.

“I don't know where I’d go to have this conversation to say what is it that I should be doing for CHILD given that he's got DLD and he's got these worries. Speech and language therapy don't think they’ve got much to offer, CAMHS batted it back, so I don't know where to go with that.” – Parent 3

It is important to note that parents were also themselves taking active steps to support their children's mental health and emotional development. Some parents explicitly sought to educate their children about mental health and their own emotions, focussing on the appropriate vocabulary and how they could communicate to others when they were struggling and needed support. This role often developed from a combination of worry for the future and lack of support from services and their child's school, and also drew upon some of the professional backgrounds of our parents.

## Discussion

This study aimed to explore the experiences of parents of children with SLCN when accessing mental health support for their children. The second stage of the study focussed specifically on the experiences of parents of children with DLD, a diagnosis which remains poorly understood or recognised by the general public.

Considering the experiences of parents of children with SLCN more widely, we found that parents’ positive and negative comments could be linked to relational and organisational aspects of their care, and professionals’ knowledge. Parents wanted to feel listened to and their concerns taken seriously, and their children supported to feel safe and confident. They wanted expedient access to appropriate mental health services and were frustrated by having referrals to CAMHS rejected, with many reporting they felt there were no services suitable for their children. They wanted the professionals that supported their child to have suitable knowledge of SLCN, and for services to work in a more joined-up manner.

The findings from the interviews of parents with DLD compliment the findings of the wider survey of parents of children with SLCN, in that these parents also reported feeling their concerns were downplayed by professionals, feeling that professionals often lacked sufficient knowledge about language needs, and reported feeling that their children did not have an appropriate service that could cater for both their language needs and mental health problems. Parents’ reports suggested that their children's language needs impacted on the mental health support they received, from the initial detection of problems in the first place, through to the delivery of treatments and interventions. Poor understanding of DLD was felt to affect the accessibility of interventions: concepts were too complex, and the settings in which the professionals worked with the children were often felt to be daunting to the child. The parents reported that in some cases, poor professional knowledge about language needs threatened therapeutic alliance, leaving children feeling misunderstood, and/or practitioners seeming to view children as uncooperative.

These findings are quite comparable to those of previous studies on mental health support for autistic people. Similar to these previous studies (Adams & Young, 2021; Camm-Crosbie et al., 2019; Read & Schofield, 2010), the present study found that families reported frequently meeting a lack of professional awareness and knowledge about their children's condition, and interventions that did not consider the children's communication needs were at best ineffective. In some cases, it seemed unadjusted interventions disrupted good therapeutic relationships forming, and children were reported to not feel safe or welcomed in these spaces.

Parents tended to view schools as places that could exacerbate or mitigate children's mental health problems, and the extent to which children were able and supported to form and maintain positive peer relationships was important to parents. The role of peers also speaks to previous work on potential protective factors for outcomes in DLD. Prosocial behaviours (sharing, caring, and being helpful) are associated with fewer concurrent (Toseeb & St Clair, 2020) and subsequent (Toseeb et al., 2020) mental health difficulties in children with DLD. Additionally, competency in social play is also associated with fewer subsequent mental health difficulties (Toseeb et al., 2020). Both prosocial and play behaviours are likely to involve children's peers.

These findings lead us to suggest several ways in which care could be improved for children with language needs. Firstly, parents reported having referrals to CAMHS rejected on the basis of their children's language problems. It should be noted that as the project only considered parent report, and did not examine administrative data or consult CAMHS clinicians themselves, we cannot know whether there were any additional reasons for why a young person with SLCN was rejected from CAMHS. Nonetheless, taking these reports at face value, we should question why the presence of a known SLCN appears to lead to CAMHS rejections. Potentially, given reports from parents that they were concerned about their children's ability to communicate distress, these children may be being rejected because their mental health needs are not easily identifiable with standard measures, or, in the context of a known SLCN, children's language problems rather than their mental health issues are seen as their main need (this could be considering a form of ‘diagnostic overshadowing’). One recommendation is that when considering the mental health needs of children with SLCN, it is important to consider how the child's language problems may be acting to hide or mask the extent of the mental health problem. Of course, all families who do not have a referral accepted to CAMHS should ideally be signposted to other appropriate support (while a child's mental health needs might not reach a sufficient threshold of CAMHS support at the time, a referral indicates significant concern around a child's mental health). In the present study, many families rejected from CAMHS felt left with nowhere to go with regards to their children's mental health. We recommend considering what services or supports for child mental health could be established with children with SLCN in mind, in order that families have resources and support to turn to if they are deemed not suitable for CAMHS.

Secondly, mental health professionals could benefit from specialist training in SLCN, ideally with specialist support from SLTs to help advise and support CAMHS clinicians in identifying children with unrecognised language needs, and to modify their approaches to make them more accessible and effective. Parents may not expect teachers and mental health professionals to be SLCN or DLD experts, but poor awareness and understanding of children's language needs was reported to negatively affect the delivery and effectiveness of interventions. Importantly, the parents in this study who were interviewed reported their children did have their language needs already diagnosed when accessing support from mental health services; yet often in mental health services, there is a high rate of unrecognised language problems (Cohen & Lipsett, 1991; Hollo et al., 2014). Thus, practitioners need to be equipped and supported to recognise the signs of language problems, so that children with unrecognised needs can be detected.

Our third recommendation concerns the delivery of mental health support. Engaging in ‘face to face’ conversations about potentially difficult emotional topics (especially without supplementary visual aids or additional activities) may be challenging and stressful for children with language needs. Many children with language needs will have experienced difficulties in holding conversations, where they have been misunderstood or felt confused. It is important therefore, that when accessing mental health support, particularly in the early stages of the therapeutic relationship, to integrate other activities that allow therapists to build rapport, reduce the demand upon language, and take an individual child-needs-led approach to the delivery of an intervention. Whilst all mental health clinicians would likely advocate this way of working with all young people referred into CAMHS, it is especially important for children with communication needs. Additionally, clinicians could be supported in making use of tools, such as apps or pictures, that could help children explain how they are feeling, without relying heavily or solely upon their spoken language skills.

A fourth recommendation is to invest in ways to support children with language needs to develop and sustain meaningful friendships. Loneliness was something that came up in several interviews, although other parents acknowledged that for their children their friendships were a key source of support and happiness. Helping children develop and maintain friendships could involve reflecting upon how children with DLD or SLCN are being removed from classroom activities, such as when receiving targeted support. Such removal might impact on children's abilities to make and retain friends and consideration of how to best support these youngsters within an ‘inclusive’ framework is vital. Indeed, evidence suggests that children with DLD who lack positive social experiences make poorer gains in social and emotional development: essentially, children who are already at a social disadvantage due to their language needs may miss out on social experience that could help them gain social and emotional skills (see van den Bedem et al., 2019).

The findings of this current study also suggest multiple avenues for future work. Firstly, research on the presentation of mental health problems in these groups, with a view to improving recognition and identification is important. In particular, research into how to detect mental health distress and enable children and young people with language needs to communicate distressing events would seem particularly urgent, given that we know these groups are at an increased risk of mental health problems, and indeed sexual exploitation (Brownlie et al., 2007) and bullying (van den Bedem et al., 2018). Systematic exploration of what adaptations improve the accessibility and success of mental health interventions for children with SLCN or DLD is clearly required. From the current results, many parents could be active and effective allies in delivering interventions, if suitably supported and guided in their role.

Future research would also need to take into account the limitations in the current project. The study had hoped to hear from a much larger sample of parents, in order to ensure the findings would be more representative, and to explore the differences and similarities of feedback from parents of children with different SLCN. The final sample size did not allow exploration of whether similar or dissimilar comments and ratings of satisfaction were being made by (for example, parents of children with DLD vs. parents of children with autism, or parents of children with specifically speech problems vs. parents of children with global intellectual difficulties, or indeed children of different ages and genders). Nonetheless, the findings of this smaller sample did resonate with previous research on the experiences of autistic children and young people. Another consideration for future research is that the interviews only included parents of children with a diagnosis of DLD. There are many more children with mental health difficulties who also have undiagnosed language problems. Indeed, it is notable that many of our parents in our interview stage came from professional backgrounds in health or social care or education: when asked, these parents said they felt their professional knowledge gave them insight into the system of getting help for their children, a privilege that not all families have (for more consideration of the issues of socioeconomic status and access to support in the context of DLD see McGregor, 2020).

Finally, future research should seek to understand the perspectives of the children and young people, as the priorities and concerns of parents may not align with those of the young people themselves. However, researchers will need to consider how best to engage with and obtain the insightful experiences and view of these children, making the research accessible whilst acknowledging their communication needs. The perspectives of professionals, such as those working in CAMHS, are also needed to understand how these children are perceived and supported, and what professionals see as barriers in services’ abilities to support children with mental health needs and SLCN.

## Conclusions

This study collected the views and experiences of 74 parents of children with SLCN via an online survey, and 9 in depth interviews with parents of children with DLD. The results suggest parents of children with language and communication needs often face a lack of understanding about their children's challenges and struggle to access services. Language problems, and professionals’ lack of understanding about these language needs, appeared to interfere with the detection of emotional distress, and with treatment and support. Greater research that supports evidence based practise in supporting children with SLCN (including DLD) to have good mental health outcomes is needed, in particular what adaptations to current practise would make services more accessible for children with language needs.

## Supplemental Material

sj-docx-1-dli-10.1177_23969415221101137 - Supplemental material for Supporting the mental health of children with speech, language and communication needs: The views and experiences of parents Click here for additional data file. Supplemental material, sj-docx-1-dli-10.1177_23969415221101137 for Supporting the mental health of children with speech, language and communication needs: The views and experiences of parents by Hannah Hobson, Mya Kalsi, Louise Cotton, Melanie Forster and Umar Toseeb in Autism & Developmental Language Impairments
