# Supplementary material for: Supporting the mental health of children with speech, language and communication needs: The views and experiences of parents
Source: Autism Dev Lang Impair. 2022 May 29;7:23969415221101137. doi: 10.1177/23969415221101137 (PMC9479119; doi:10.1177/23969415221101137)
Supplement: sj-docx-1-dli-10.1177_23969415221101137 - Supplemental material for Supporting the mental health of children with speech, language and communication needs: The views and experiences of parents [file sj-docx-1-dli-10.1177_23969415221101137.docx]

| ***Supplementary Table 1: Additional supporting quotations from interview participants*** |
| --- |
| **Language needs affect detection and presentation of distress** |
| “*…Afterwards he was in the car and he was very distressed and we were talking about it and he said on the way there he felt suicidal, which I would’ve never have picked up on at all. … And I suppose that’s my concern more around his mental health because sometimes I think there’s risk linked to that because I think people might not understand how severely he’s feeling because he’s not able to express it, and because we don’t ask um, check in because he’s not giving um verbal signals*” – Parent 1  “*So, when he was really wee, well he wasn’t actually really wee, he just didn’t have any language, and he just would refuse to go to sleep at night but he was just scared – felt really scared and he couldn’t say that. It wasn’t until like a year later – a full year of every night time being a battle, that he was able to point and go say ‘monster’ and there was a shadow and he thought it was a monster. Now, if he’d have been able to tell me that a year earlier, I could’ve fixed it*.” – Parent 3  “*Sometimes it can be the identification of that problem that’s the issue– and knowing it’s a problem, rather than just accepting it, that that’s happening to you. So yes, if it’s something that you can identify and understand and therefore know that it’s- it’s not normal what’s happening or as an issue then yeah, but quite often it’s that identification of what’s happening to me, do I know what’s happening to me. It’s that sort of processing of your emotions really, or the scenario to know actually, it’s not right and therefore I might need a bit of advice on that. And if he gets to that situation then yes, absolutely he would, but then sometimes I’m not sure in the past that that’s actually even been registered.*” - Parent 9 |
| **Traditional approaches may not be appropriate for DLD** |
| “*I thought play therapy because it’s not verbal, or because it’s not based on verbal and it wouldn’t be obvious to him. Like if I sat down, like we’re going to talk about this, he’s just worried, whereas playing, it’s playing I think it’s a level playing field, and I think a good play therapist would allow him to feel safe and in control and explore the worries in a way that feels safe to him. Art therapy or a non-verbal kind of therapy where he just could be and feel a sense autonomy and that he wasn’t on the back foot a little bit – that was my thinking*.” – Parent 3  “*It* [an app] *has faces one to ten and each day he has to put where he is on that, and if he gets above a certain number he has to tell staff – he was learning to cue staff in and self-monitor.”* – Parent 1  “*They* [professionals] *need to think about understanding – about their* [children’s] *understanding about their emotions, you know, it needs to be visual and they need to be talking about their emotions and different emotions – how to express them in what’s appropriate socially.”* - Parent 7  “*Some of the reasonings and the language that they were saying, you know, like they had the emotional ladder and where they were on the ladder to be able to get them to say school, you know, I still felt like they were using quite complex concepts for CHILD to understand. CHILD didn’t have that understanding… So I was constantly having to re-adapt, particularly their strategies at CAMHS, definitely, they were far too complex for him*.” – Parent 6  “*I already know from having worked in CAMHS and been in the system there was no way they were going to take that on, it was just nowhere near their bag. But also even if they did take it on, I don’t think they’d have been able to take account for his language difficulties – they might’ve had it in their heads as being important, but I don’t think they could have adapted their interventions adequately without a speech and language therapist. And certainly our local CAMHS teams don’t have speech and language therapists in them*.” – Parent 3 |
| **The role of school environment** |
| “*I remember she started crying saying she didn’t want to go to school and we’d never had that with her before... But I think what really upset her was that she was being kept in at breaktime because she couldn’t finish the work, you know, and that for her - it’s a big release going out to play for any child, but for her she really needed that... So, I think she was just generally getting fed up and shouted at and being told she wasn’t listening*” – Parent 4  *“So she [SCHOOL PROFESSIONAL] brought him packs, she came into school or to here and she worked with CHILD and they went through a pack about getting ready for secondary school, and they talked about emotions and feelings, and how he could deal with them, what they could feel like. …Sort of like things like what would happen at school, what it might look like how would you get there – all little things that you may not think about she all planned out for CHILD so I think he was really prepared for secondary school because of that.* – Parent 7  *“…he wasn’t allowed to see his friends, because I was told his friends have moved on, so he couldn’t have contact with, you know, he wasn’t allowed – they kind of put him in a- the learning zone support place but made – he wasn’t allowed to go into the sort of support anywhere with the mainstream kids and I don’t know. Yeah, it was very tough.”* – Parent 8  *I was like can you please differentiate the work for her and then I got told use year 1 on the website, which wasn’t brilliant either because you’re not helping her to perhaps talk to her friends when they have all the zooms. You know, we’re doing the jungle and then you’re not doing the jungle, so you’re already at a disadvantage, you can’t participate. So, I think it was awkward.* – Parent 5  “*The response was pretty much we can’t micromanage friendships, we can’t tell children to play with other children, which having two older children, I completely understand that. But at the same time, there are children that are vulnerable to not making friendships, and they do need support…”* – Parent 2 |
| **The role of key professionals** |
| *“I do remember the SENCO saying to me – “I know your friend has” - you know, she used the work “friend” – “I know your friend has diagnosed her but I still think there is something else”. Well I said that’s fine, if you think there is please assess, do whatever you need, but I don’t think so um. And then I’ve not heard anything back from that.”* – Parent 4  “*I was trying to persuade the SENCO that we needed extra help emotionally for him, and again she was fobbing me off until he was in year 5, and they were seeing a lot more extreme behaviour at school, so they were starting to believe me a little bit more about how bad it was.”* – Parent 6 |
| *“And support for staff, I think it’s not always just about supporting the child - if there was someone going into CHILDs school talking about DLD, telling them about what it is, making it very easy for them to know what it is, because I know they haven’t got lots of time, but being present and being able to give lessons and then give advice about how she could’ve changed that lesson to make it more accessible.”– P4*  “*Well, I’ve struggled really because the speech and language team that we’re involved with are amazing but they’re not mental health professionals, and when I’ve kind of approached them for advise you can just fort of see the look on their face like oh I don’t know what to do about that.”* – Parent 3  “*I get on well with our GP, she’s lovely but she’s quite useless, and erm she pushed me back to the school, and the school pushed me back to the GP, so I was like a ping pong going like this* [back and forth]*.*” - Parent 6  *“I was frustrated… I think they didn’t do- possibly because they can’t do- some of the things I was wanting them to do, that I would’ve done. So, there’s some things I would’ve wanted him to do – I wanted them to do things around um – but I had to suggest some of the things, I think, that was my frustration” – P1* |
| **The role of and impact on parents** |
| “*We talk a lot about emotions and feelings and naming them and how that feels and what that means, so I think that he’s just really got into his stride about being able to say I feel this and I don’t really know why. That definitely helps but… So in some ways, I suppose what I’m saying is that I don’t think he would have these worries without the DLD, but also because of his DLD I have been much more on it about helping him work through and understand them, which we might not have been able to do with my older child so it contributes a lot.”* – Parent 3  “*So we have had that sort of discussions with him about – it’s more trying to give him strategies to manage the distress it causes him in the moment, if he can’t process quickly enough when he’s distressed or upset, that’s the difficulty*.” – Parent 1  “*I don’t know a single other person with a child with DLD, not one – I mean I must do, but I don’t know them*” – Parent 3  “*We’d have never have got that diagnosis if I didn’t have this job, or even had a colleague that was specialised in it – and what’s the chances of that, you know.”* – Parent 4 |
